# Breeding Services and the Factors Influencing Their Use on Smallholder Dairy Farms in Central Uganda

**DOI:** 10.1155/2014/169380

**Published:** 2014-03-17

**Authors:** Anthony Mugisha, Vincent Kayiizi, David Owiny, John Mburu

**Affiliations:** ^1^College of Veterinary Medicine, Animal Resources and Biosecurity, Makerere University, P.O. Box 7062, Kampala, Uganda; ^2^Faculty of Agriculture, University of Nairobi, Kenya

## Abstract

Dairy cattle breeding is an important technology in the enhancement and promotion of dairy production in Uganda. The introduction of germplasm through AI is crucial to enhance the production potential of the local breeds. A study was conducted in six districts of Uganda in the central region using a questionnaire survey involving 450 randomly selected households to profile the dairy breeding services in use and investigate the factors that affect the success of dairy breeding focusing on AI. Adoption of the AI service was highly (*P* < 0.05) dependent on ava ilability of extension services, record keeping practice (*P* < 0.05), and availability of milk markets (*P* < 0.05). On the other hand AI adoption was independent of formal education, age of farmer, labor availability, and feed/water availability (*P* > 0.05). Use or nonuse of AI did not significantly (*P* > 0.05) influence the sex of the calf born. While preference for AI was marked, very few farmers actually used it. This implies that focus should be put on improved AI service delivery alongside improved extension services.

## 1. Introduction

In Uganda, most of the dairy breeds are kept on smallholder units, keeping about 10 animals or less [1]. Although animal genetic improvement offers one of the most efficient and quickest ways of improving the productivity of dairy herds, its effective exploitation has not been achieved due to lack of a well planned and executed breeding program despite the unrelenting attempts to upgrade the national dairy herd [2]. The Uganda Bureau of Statistics (UBOS) estimates improved dairy breeds (exotics and crosses) to constitute only about 6.4% of the national cattle herd [3]. The genetic constitution of most of the grade animals, however, is not precisely known due to the indiscriminate cross-breeding without proper records [4]. This has led to wastage of valuable genes as the offspring of superior dairy cattle is not made good use of. Dairy production also faces the multipronged farming objective of most dairy farmers where other livestock values, some of which noneconomic, override the milk production value of the animals kept [5]. This is expressed in the choice of breeds and the breeding system that downplays the importance of milk production traits.

The prevailing market forces favor high milk producing cattle especially near major urban centers [6]. In such areas land use patterns have changed. The urban market of the elite population with favorable purchasing power, food preferences, and habits is set to increase by 25% by 2025 [7]. This land use change should be followed by keeping a few but high grade animals that can ensure adequate returns, with breeding practices geared towards genetic improvement. It also puts a limit to uncontrolled breeding and opens avenues to exploiting improved breeding systems.

At farm level, certain qualities, characteristics, or traits are sought after in the breeding stock. They are those that best satisfy the interest or best suit the farmers' situation and objective. Since the farmer is not in isolation but part of a production system, the availability and accessibility of the desirable genes and their distribution mechanisms (breeding services) are determined by certain factors. These affect the breeding decisions at farm level and could influence the growth of the dairy genetic pool and smallholder dairy production as a whole.

Historically, in Uganda, use of* Bos indicus* breeds normally dominates the free grazing systems as opposed to* Bos taurus* breeds which are dominant in the more intensive systems coupled with extensive use of bulls indicating possibility of inbreeding [2, 5]. Use of improved breeding services like artificial insemination (AI) also follows the same pattern [8].

Until the 1950s, efforts to improve dairy production in the country were based almost entirely on selective breeding within indigenous cattle with fear that exotic breeds were likely to be adversely affected by the prevailing climate (save for the high altitude regions). Realizing that selective breeding with the local cattle population would be too slow to match the rate of development of Uganda's agriculture, introduction of European-type cattle and AI of indigenous cattle with exotic semen was started in 1959 [4], and since then there has been continued importation of semen. This led to an increase in the improved cattle population in Uganda from apparently none in 1958 to about 18,000 in 1969 [9], and from 209,000 in 1994 to 279,000 in 1999. However, semen importation has been driven by production and not linked to a meaningful breeding programme [10]. Consequently, the growth of stock of exotic and improved herds has not been great.

A variety of dairy breeding services are currently available to farmers, ranging from natural service to AI. An important consideration is whether the farmers choose the service or they are constrained in their choice. Although availability of improved breeding services is key to sustained dairy development, access to AI in Uganda is reportedly low, averaging between 2% and 15% [1], and this is probably concentrated mainly in the central region. Staal and Kaguongo [1] attribute the suboptimal use of AI to low availability, high cost, and uncertain reliability. In 1997, a two-year ban was instituted on importation of animal products and genetic materials including semen and embryos due to the bovine spongiform encephalopathy (BSE) scare, creating a need for local semen production. This, however, has met challenges of lack of progeny tested bulls owing to an inconsistent herd recording programme.

This study thus sought to assess the factors that influence the use of dairy breeding services in the region, with the specific objectives of identifying the most desired types, breeds, traits, and qualities of dairy breeding stock in the study area; profiling the prevalent dairy breeding services available to the farmer and determine their level of use; and assess the factors/constraints to improved dairy breeding in the study area.

## 2. Methodology

The study covered five districts in Central Uganda, namely, Mukono, Kayunga, Luweero, Nakasongola, and Masaka districts. The predominant farming system in this region can be summarized as an intensive banana-coffee system [11], and dairy farming is mainly practiced in these areas on a small scale.

The study area experiences a bimodal rain pattern separated by two relatively short dry seasons running from December to March and June to July [11]. These conditions however vary over the study area, with some parts experiencing a longer dry season than others or periodic severe droughts. These drier areas are part of the Ugandan cattle corridor that traverses the north-western part of the central region.

The study was a survey designed to collect data on dairy breeding. The research team visited each of the five study districts, interviewing farm household heads in the specified locations using questionnaires. In order to ensure that the collected data were representative, the following procedure was followed to establish the minimum sample size.

The standard procedure in obtaining the sample size applied the following formula [12]:

(1) $$\begin{array}{c} Y={\left[1.96*\frac{\text{SD}}{\text{ME}}\right]}^{2}, \end{array}$$

where *Y* is the minimum sample size; SD is standard deviation; ME is margins of error, and at 95% confidence interval (1.96). According to a previous study in the context of a smallholder dairy development, standard deviation of milk production per cow was 4.3 kg [13]. This value was substituted in the above formula together with a marginal error of unity. Accordingly, a minimum sample size was calculated as 71 households per study district, but this was increased to 75 to simplify enumeration in the field and allow for incomplete data.

In all survey districts no sampling frame was available. In the circumstances, a geographical random sampling [14] proved to be most suitable. First, each survey site was defined as the hub catchment area-a circular area of 20 km, with the hub at the centre of the circle irrespective of administrative boundaries. The corresponding radius in each case was chosen based on the maximum feasible distance farmers or traders would travel to supply milk to the chilling plants. The circular survey area was divided into grid cells which, depending on population density, averaged about 85 square meters. In all cases, urban, unpopulated areas, forest, and marshy areas were masked out. Finally, by applying a simple random sampling technique, 75 grids were selected from all the grids by assuming that the area of each grid equates approximately to an average homestead area of one farm household.

The process of identifying respondent households and approaching the interviewees for the survey involved the following procedure: the centre of each of the 75 grids was assigned a latitude and longitude coordinate which were then uploaded into a global positioning system (GPS) hand set. The survey team guided by a GPS handset went to each location and administered the questionnaire to a household situated nearest to the grid referenced point in that particular grid cell. When the survey team encountered more than one household in the grid cell and the coordinate located in between, then the team would randomly select one of the households. When there were no households in the vicinity of the GPS coordinate, then the survey team would randomly select a direction (north, south east, or west) and walk guided by the GPS/compass until a farmhouse was found.

The data obtained was mainly quantitative in nature. It was categorized and captured in Microsoft Access software before analyzing using the STATA* package. The different findings were related to the level of use of the different dairy breeding systems using the statistical procedures. Data was analyzed at two levels: the univariate and bivariate levels.

At the univariate level, simple frequencies and their percentages were obtained and that was used to analyze background characteristics of the study. At the bivariate level, cross-tabulations were done and the Chi-square analysis was carried out. Artificial insemination was considered the dependent variable and other selected factors were considered independent variables. The Pearson Chi-square test was used to show the level of association when the threshold *P* value is set at 0.05. For the probability value less than or equal to 0.05, it was concluded that the relationship between use of AI and independent variable is statistically significant.

To supplement the data obtained in the questionnaire survey, focus group discussions were held in each district comprising a minimum of 20 farmer participants per district.

## 3. Results

This study found that indigenous types (Nganda, Karamojong Zebu, and Ankole) comprised 69% of the cattle population. Holstein Friesian and their crosses constituted 17%, Boran 11%, and other breeds accounted for only 3% of the cattle population.

The overall average age at first calving was 36.4 months, while the average number of services/inseminations required per conception was 1.4. The average calving interval was 14.4 months; average total milk production at calving was 3.5 liters, while average total milk production the day before the interview was 1.9 liters (Table 1). About half, 49.4% and 50.6%, of the calves borne were male and female, respectively, and 84.9% of the calves were present on the farm.

In the focus group discussions, criteria employed by farmers in selecting breeding stock were highlighted. For dairy purposes, the following traits were related to superior milk production according to the farmers: wide arched back, long teats, small/short neck, prominent milk vein, well placed and big udder that is not sagging, high milk yield of the mother of the bull, big naval, strong and straight legs, loose fleshy thighs, and for bulls, large testicles.

Majority of the respondent farmers (63.6%) preferred bull service to AI. In-depth interviews indicated that farmers did not have many options in sourcing for breeding services. Bulls/natural service was reported to be the main service available. Other reasons given as to why AI service was not preferred were the notion that AI purported to produce more bull calves than females, lack of money to pay for AI at the time when animals are on heat, AI services being quite far from farmers and the perception that they are expensive, lack of sensitization about the benefits of AI, very high expectations of AI service which are rarely met, higher chances of dystocia in cases where AI was used coupled with limited number of veterinary practitioners to attend to the dystocias. Farmers argued that with natural service, bull owners did not charge money for repeat services which was not the case for AI.

Although the larger proportion (56%) of the farmers indicated preference for AI, only 7.2% actually used the service. Farmers reported that more problems were experienced with AI service (69.2%) as compared to bull service (46.9%).

In most of the cases, AI services were provided by private practitioners and NGOs, and government played a less significant role, providing only 19% of the AI services.

From Table 2, it is apparent that AI service was five times more expensive than natural service.

### 3.1. Relationship between Selected Factors and Improved Dairy Breeding (AI)

Some household socioeconomic and demographic conditions had a relationship with the use of artificial insemination as an improved dairy breeding service (Table 3).

There was no association between use of AI and the gender of the household head (*P* = 0.56), age of the household head/farmer (*P* = 0.57), education level of the household head/years of schooling (*P* = 0.89), involvement in community leadership of the household head (*P* = 0.87), availability of water (*P* = 0.34), type of labor (family or hired) used (*P* = 0.14), and access to credit for dairy activities (*P* = 0.13). While this was the case, the study showed a strong inverse relationship between AI use and size of grazing land (*P* = 0.00). The farmers holding small land sizes were more likely to use AI. A direct relationship was observed between use of AI and availability of extension services (*P* = 0.01), record keeping practice (*P* = 0.00), availability of milk markets (*P* = 0.01), and preference for AI service (0.00). An interesting finding was the difference between perceptive preference and actual use, where only 18% of those who had perceptive preference for AI actually used it. The rest used bull service.

On the other hand AI was not found to influence the sex of the calf born. This was in relation to the most recent calf borne (*P* = 0.46). However, univariate analysis showed that slightly more males were born from AI service than with natural service (see Table 4).

### 3.2. Feed and Water for Dairy Production

In the past, the main system for keeping cattle was almost entirely by grazing during both rain and dry seasons. At the time of the study which was in the rain season, grazing was practiced by 84% of the respondents; and in the dry season it was 82.4%. The other system for keeping cattle was grazing coupled with some stall feeding. Majority (85%) of the farmers reported experiencing a shortage of feeds, especially during the dry season.

## 4. Discussion 

Natural service remains the major method of dairy breeding with over 90% of the respondents reporting it to be the service used in the previous 5 years. More farmers preferred AI (36%) as compared to those who actually used it (7.2%). This reveals a strong contrast between preference and actual use of the service. In Kenya, Baltenweck et al. [8] attributed such a situation to low availability/access, high cost of the service, and technical failures that led to many repeats. It also represents a considerable decline in the use of the service, as past studies indicated that central Uganda rated higher (15%) in the use of AI [1]. However, the findings contradict earlier reports of a positive trend in the number of inseminations per year from NAGRC and DB, which may be a case of misreporting by the AI field staff. The perceived high cost of AI relative to natural service, and the farmers' unmet high expectations of AI, could also be important reasons for high preference but low utilization of AI services. The high expectations may include overestimating the heritability of milk production traits, expecting more female calves than can be scientifically justifiable.

On the farmers' attitude towards AI service, farmers believed that AI produced more male calves than females. The findings seemed to support the farmers' claims and showed that the proportion of males among the most recent calves on the farms visited was 49.53% where natural service was used and 58.82% where AI was used. However, the relationship was not statistically significant (*P* = 0.46). The observed higher proportion of males in AI could be related to timing. Pursley et al. [15] showed that in oestrus-synchronized cattle cows that inseminated early (0 hrs) and late (32 hrs) had higher proportions of producing female calves, but with less conception rates than those bred in between these times. In principle, technicians chose to carry out AI at times that ensured maximum conception rates rather than targeting proportions of male or female calves.

The leading actors in provision of AI service were private service providers and nongovernmental organizations (NGOs). The minimal involvement of government is probably due to the policy shift where such services are now classified as private [16]. Ngigi [17] noted that government subsidies on AI were responsible for the growth of dairy production in Kenya, and their removal resulted in a decline in use of the service. It is probable that the decline in annual inseminations noted in the study area is due to the reduced government involvement in the provision of these services, coupled with a weak private sector. This is likely to be the trend for AI in developing economies that have embraced structural adjustment policies. Breeding programs therefore should be redesigned to suit the changing macroeconomic policies in order to sustain and improve the past achievements in dairy breeding.

There are quite a number of farmer-related factors that were considered to relate to the rate of adoption of AI. The study noted a strong inverse relationship between AI use and size of grazing land, whereby the farmers holding small land sizes were more likely to use AI (*P* = 0.01). This is due to the fact that such farmers want to maximize their investment through intensification, and the small number of animals kept on such holdings does not justify keeping of bulls. The zero grazing system is typical of this. As a breeding technology, AI is also more economical in the small compared to the large holdings in terms of direct costs [18]. Bigger herds and grazing land present more hardships in heat detection and timing, resulting in reduction in conception rates. Since most of the farmers were smallholders, on small pieces of land, they were in a better position to utilize AI, other factors remaining favorable.

Although small land holdings correlated with higher likelihood of utilizing AI, Balikowa [19] indicated land scarcity as one of the constraints to dairy farming. This is true because the benefit of this land-AI use could be short term and within limits. The farmers need guidance on the breeding services that best suit the size of their holding and the level of production. Thus, extension services have to be customized to the specific situations of farmers.

The task of agricultural extension appeared to be shared between government, private practitioners, and NGOs. The issue here is whether the messages the farmers receive are unified and not biased by commercial interest of the private practitioners, for sale of their drugs and services. Availability of extension services was found to strongly correlate with AI adoption (*P* = 0.001). Similarly, Kaaya et al. [20] found extension services to be crucial for AI adoption. Although Wambugu et al. [21] found that most poor farmers rely on neighbours as their primary information source, extension is generally believed to influence the farmer's preference for certain breeds and affects better farm management practices, which among others include record keeping practice that was highly related to AI use (*P* = 0.00). On the other hand, AI can also be viewed as a precursor for demand for continued extension services, as the improved dairy breeds require more extension knowledge and farming skills. The extension messages in turn result into preference for improved breeding technology (AI) (*P* = 0.00). This is why extension services, record keeping, and preference for AI were positively correlated with AI. The radio as a source of information ranked highest (80%). This is therefore an important channel for extension programs to create awareness and conduct actual training of farmers on dairy breeding and other production practices and techniques.

The practice of record keeping is very central in the management of any breeding program yet it is still a major constraint in this country [4]. The study found that only 22% of the farmers kept meaningful records, and these invariably had a high propensity to use AI. According to Omore et al., [22], generally most smallholder farmers do not maintain records. Balikowa [19] found that only 14% of the farmers in south western Uganda kept meaningful records and this should be the main focus not only for breeding programs but also production and marketing interventions.

Majority (61.7%) of the farmers did not sell milk indicating that milk production is still at subsistence level. The reasons to this could be difficulties in milk marketing including low and sharply fluctuating milk prices that follow seasonality in quantity of milk produced, especially in rural areas. The study noted a strong association between the use of AI with availability of milk markets (*P* = 0.01), a finding similar to that of Kaaya et al. [20], probably because the latter serves as an incentive for technology adoption as well as continued investment [23]. Better organization of the informal sector and strengthening of the formal sector in addition to other critical inputs like feeds and water as incentives would contribute significantly to adoption of AI as a dairy breeding technology.

There was reported scarcity of feed and water in the study area, especially during the dry season. Most farmers could afford to water their animals only once a day, and only a few could afford constant water supply irrespective of seasons. However, the study noted no statistical relationship between AI and availability of water (*P* = 0.34). Muli et al. [24] reported that availability of water may have no direct relationship with the amount of milk produced. The incidence of feed shortage (reduction by 84%) has probably affected the success of AI. Since feed is a main cost factor in dairy production [25] making up to 60% of total daily production cost [26], this finding may be an indication of change of farming objective or gradual exit from dairying, in favor of the more feed-tolerant and resilient nondairy/multipurpose breeds or even off-farm enterprises.

In the USA, Bragg and Dalton [23] indicated that older farmers were more likely to quit dairying. Similarly Kaaya et al. [20] also indicated age as negatively associated with use of AI in central Uganda. This study however found no association between use of AI and the age of the farmer or the household head (*P* = 0.57). Generally, older farmers would be in better position to undertake long term stable investments that may not be attractive to the youth. One would also expect that educated farmers would be more likely to use AI due to their propensity for adoption and agricultural productivity (Koma, 2003; [16]). On the contrary, the study found no relationship between education level of the farmer and use of AI (*P* = 0.89), probably because highly educated farmers only do dairying as a secondary activity and lack the necessary concentration demanded by AI services. The relationship between use of AI and education if it exists could only be within very limited ranges of basic education that were not defined by this study. Otherwise in general terms, adoption aptitude of the farmer for AI was independent of formal education.

## 5. Conclusion

Natural service remains the breeding method available to most smallholder dairy farmers in Central Uganda, although a considerable percentage of the farmers indicated apparent preference for AI. The size of grazing land (smaller holdings), practice of record keeping, preference for AI, availability of milk markets, and access to extension services are key factors that determine the success of AI services.

There was no significant relationship between use of either AI or natural service and the sex of the calf borne. This therefore dispels the farmers' claims that AI produces more bulls than female calves. The level of education of a farmer is not a significant factor for the adoption of AI by the farmers in Uganda.

## Figures and Tables

**Table 1:** Average reproductive performance of dairy and nondairy breed cows in the herds.

Cow characteristic	Age at 1st calving in months	Number of Services per conception	Calving interval in months	Milk production in liters at calving	Milk production in liters (previous day)
Dairy breed	29.7	1.7	13.1	6.9	3.9
Nondairy breed	38.0	1.3	14.7	2.7	1.5
Overall average	**36.4**	**1.4**	**14.4**	**3.5**	**1.9**

**Table 2:** Cost of breeding services previously used.

Expenditure	AI service	Bull service
Total expenditure in the last 12 months (United States Dollars)	19.10	14.40
How much paid for the last service (United States Dollars)	11.10	2.30

**Table 3:** Relationship between use of AI and socioeconomic factors of the farmers.

Characteristics	% of used AI (*n* = 14)	% of never used AI (*n* = 180)
Availability of hired labor for dairy		
No	9.9	90.1
Yes	4.4	95.6
Education/years of schooling		
0–6	6.36	93.64
7–13	8.11	91.89
14–20	12.5	87.5
Above 20	0.00	100.0
Keeping records*		
No	1.37	98.63
Yes	24.32	75.68
Access to credit for dairy services		
No	6.18	93.82
Yes	18.18	81.82
Availability of milk markets*		
No	3.31	96.69
Yes	13.89	86.11
Preference for the use of AI*		
Yes	18.75	81.25
No	3.49	96.51
Availability of grazing land (acres)*		
Small (≤5 acres)	11.88	88.12
Big (≥6 acres)	2.22	97.78
Access to extension services*		
No	1.39	98.61
Yes	18.46	81.54

*P < 0.05; based on Chi-square.

**Table 4:** Relationship between use of AI and the sex of the calf born.

Use of artificial insemination	Sex of the most recent calf		
	% of male	% of female	Total
Never used	49.53	50.47	100
Used artificial insemination	58.82	41.18	100
